# Development of a straightforward direct injection UHPLC-MS/MS method for quantification of plastic additive chemicals in roadside retention ponds

**DOI:** 10.1007/s00216-024-05657-3

**Published:** 2024-11-25

**Authors:** Katie McKenzie, Angela Pllu, Iain Campbell, Linda A. Lawton, Bruce Petrie

**Affiliations:** 1 School of Pharmacy, Applied Sciences and Public Health, Robert Gordon University, Aberdeen, AB10 7GJ UK; 2 Balfour Beatty plc, UK Construction Services – Motherwell, Scotland, ML1 4WQ UK

**Keywords:** Tyre, Sustainable drainage system, Microplastic, TRWP, Vulcanisation, HMMM

## Abstract

There is growing interest in road pollution that enters surface waters. Additive chemicals used in the manufacture of plastics, including tyre rubber, are mobile pollutants that pose a threat to aquatic life. Therefore, an ultra-high performance liquid chromatography tandem mass spectrometry (UHPLC-MS/MS) method was developed to measure 25 plastic additive chemicals in road runoff and water of retention ponds used to manage road runoff. A straightforward direct injection methodology was adopted to minimise sample handling and risk of contamination. Using this approach, the method quantitation limits (MQLs) ranged from 4.3 × 10^−3^ to 13 µg/L. These were adequate to determine most chemicals at or below their freshwater predicted no-effect concentration (PNEC). Method trueness ranged from 18 to 148% with most chemicals being within 80–120%. The method was applied to water from four retention ponds in series to measure additive chemicals entering the ponds (i.e., in road runoff) and passing through each pond. Greatest concentrations were observed in road runoff during heavy rainfall following dry weather. Here, 1,3-diphenylguanidine (DPG) exceeded its current PNEC of 1.05 µg/L. Notably, *N*-(1,3-dimethylbutyl)-*N′*-phenyl-*p*-phenylenediamine-quinone (6PPD-quinone) was determined at 0.13 µg/L which is greater than its lowest acute toxicity threshold (0.095 µg/L). Similarity in additive chemical concentrations throughout the retention ponds during steady flow suggests little or no removal occurs. However, further studies are needed to assess the fate and removal of plastic additive chemicals in retention ponds and the risk posed to aquatic environments. Such research can be facilitated by this newly developed UHPLC-MS/MS method.

**Graphical Abstract:**

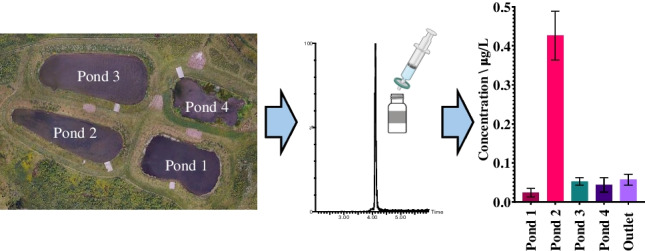

**Supplementary Information:**

The online version contains supplementary material available at 10.1007/s00216-024-05657-3.

## Introduction

There is growing interest in the impact of roads to surface water quality. Road runoff contains a cocktail of pollutants including particulate matter (e.g., microplastics—MPs) and dissolved organic contaminants (e.g., plastic additive chemicals) [1]. A major contributor to the load of MPs in road runoff is tyre and road wear particles (TRWPs) [2–6]. Synthetic tyre rubber particles are considered MPs under the broad definition of MPs being all man-made macromolecular materials [7], smaller than 5 mm in size [8]. TRWPs are formed by friction between tyres and road surfaces during driving and are a combination of tyre wear particles, brake particles, road material, and road markings [7, 9]. Importantly, plastics including tyre rubber contain a variety of additive chemicals that can leach into water and are potentially hazardous to aquatic organisms. Leaching can occur directly from tyres and macroplastics as well as from MPs generated on the road surface.

A large variety of chemicals are used during the manufacture of plastics and tyres specifically. They can make a significant contribution to the mass of the final product. For example, additive chemicals account for approximately 5–10% of a tyre’s composition [10]. Their presence in the wider environment can be an indicator of tyre pollution, however, it should be noted that they are not all specific to tyres and can have other sources. Benzothiazoles, often used during the rubber vulcanisation (hardening) process, also have uses in antifreezes and pesticides [11]. The vulcanisation process improves the durability and strength of tyre rubbers by forming cross linkages. A range of chemicals are used to facilitate and speed up the process. Chemical additives also serve a variety of other purposes in tyre manufacturing. They can be used as stabilisers, protective additives, and flame retardants. Additives associated with tyres have previously been detected in road runoff. Zhang et al. [12] detected 19 of 23 target chemicals in surface runoff samples including vulcanisation additives such as DPG and several benzothiazoles, as well as transformation products.

Several additive chemicals associated with tyres have been detected in surface waters which receive stormwater. DPG has been reported in Australia and the USA at concentrations in the range 0.013–1.1 µg/L and 5.0 × 10^−3^–0.54 µg/L, respectively [1, 13]. In Canada, a peak river concentration of 0.52 µg/L for DPG was observed following rainfall [14]. With concentrations being in the same order of magnitude as the current lowest freshwater PNEC of 1.05 µg/L [15] and lack of monitoring data, there is a need to expand our knowledge on the presence of these chemicals in the environment. Transformation and thermal degradation products of additive chemical have been found to be as harmful, if not more harmful, than their parent compound. One such product which is of particular interest is 6PPD-quinone due to its high toxicity towards coho salmon (*Oncorhynchus kisutch*), with a reported *LC*_*50*_ (median lethal concentration) value of 0.095 µg/L [16]. This is a transformation product of 6PPD, an antioxidant used in tyres and has been found to be the most abundant PPD-quinone in the water environment [17]. It has been detected in two studies of Australian stormwaters in the concentration range of 3.8 × 10^−4^–0.088 µg/L and 5.0 × 10^−5^–0.024 µg/L, respectively [1, 18] as well as in North American and Chinese waters [12, 19, 20]. In Canada, it has been reported at a peak river concentration of 2.85 µg/L following rainfall [14].

Many countries now outline management requirements for road runoff due to the concerns for the surrounding aquatic environment. For example, Scotland requires new road developments to be drained by an appropriate sustainable drainage/sustainable urban drainage system (SuDS) to avoid pollution of the water environment [8]. SuDS work alongside conventional drainage systems to drain surface water from roads [21]. Retention ponds are a common type of SuDS used for road runoff. These ponds retain the runoff for a period of time allowing for physical, chemical, and biological removal processes to reduce or eliminate pollutants before their release into the wider aquatic environment [22]. Despite many plastic chemicals having been detected in road runoff samples, there is little information on the suitability of SuDS, such as retention ponds, to remove these pollutants from water. Retention ponds also serve as important habitats for a variety of aquatic life. Therefore, it is important to measure these chemicals within the ponds and benchmark their concentrations against toxicity thresholds.

Currently, there are limited validated analytical methods described in the mainstream literature for the determination of plastic additive chemicals in water. UHPLC-MS/MS methods have been used to determine some of these organic chemicals in aquatic samples [12, 20]. However, pre-concentration or clean-up steps such as solid phase extraction (SPE) are often required to ensure suitably low detection capabilities for environmental monitoring. Targeted mass spectrometers (e.g., triple quadrupoles) are now sufficiently sensitive to facilitate trace (µg/L) environmental analysis of organic chemicals by direct injection [23–26]. Utilising direct injection analysis reduces sample preparation time, costs, and the possibility of contamination, which is particularly important for plastic-related chemicals due to the ubiquity of plastics in everyday life. However, utilising plastic syringe filters, commonly polytetrafluoroethylene (PTFE) or polyvinylidene fluoride (PVDF) membranes, is popular as a rapid means of removing particulate matter from water samples prior to analysis [12, 23–25, 27–29]. For the analysis of plastic additive chemicals, this could be a source of contamination which needs assessment. Another important consideration for the use of direct injection for environmental analysis is to assess the impact of other substances in the sample (which would otherwise be removed or reduced by SPE) that cause signal suppression during ionisation. These interferences can influence the accuracy of reported data particularly for multi-residue methods where all analytes do not have a corresponding deuterated surrogate available [25].

Considering the lack of available methodologies to monitor plastic additive chemicals in water, the objectives of this work were to: (i) develop and validate a direct injection UHPLC-MS/MS method for plastic additive chemicals at µg/L concentrations in road run off and the outlet of retention pond water samples, and (ii) measure the concentrations of plastic additive chemicals in retention ponds used to manage road runoff. A total of 25 plastic additive chemicals were investigated including stabilisers, flame retardants, thermal degradation products, vulcanisation additives, vulcanisation accelerators, plasticisers, protective additives, and transformation products.

## Materials and methods

### Materials

The analytical standards bisphenol-A (BPA), 5-methylbenzotriazole (5-MBTR), 1,2,5,6,9,10-hexabromododecane (HBCD), 1-cyclohexyl-3-phenylurea (CPU), 3-cyclohexyl-1,1-dimethylurea (C-DMU), benzothiazole (BT), 2-(4-morpholinyl)benzothiazole (24MoBT), 2-mercaptobenzothiazole (MBT), N-cyclohexyl-1,3-benzothiazol-2-amine (NCBA), dimethylphthalate (DMP), diethylphthalate (DEP), benzylbutylphthalate (BBP), and di-*n*-pentylphthalate (DnPP) were purchased from Sigma-Aldrich (Gillingham, UK). 1H-benzotriazole (BTR), *N*,*N*-dicyclohexylmethylamine (M-DCA), 2-aminobenzothiazole (2-ABT), 2-hydroxybenzothiazole (2-OHBT), and DPG were acquired from Fisher Scientific (Loughborough, UK). Tetrabromobisphenol-A (TBBPA), 2-(methylthio)benzothiazole (2-MTBT), and 9,10-dihydro-9,9-dimethylacridine (BLE) were purchased from Tokyo Chemical Industry (Oxford, UK). 2,4,6-Tris(bis(methoxymethyl)amino-1,3,5-triazine (or hexamethoxymethylmelamine, HMMM), and the deuterated surrogates BPA-*d*_*8*_ and 5-MTBR-*d*_*6*_ were obtained from Toronto Research Chemicals (TRC, Canada). Finally, 6PPD-quinone, *N*-cyclohexyl-*N′*-phenyl-*p*-phenylenediamine (CPPD), and 6PPD-quinone-*d*_*5*_ were from LGC chemicals (Middlesex, UK). HPLC grade methanol, ammonium formate, formic acid, ammonium fluoride, and PTFE and PVDF syringe filters (0.45 µm) were obtained from Fisher Scientific. A full list of the additives, their abbreviations, properties, and structures are provided (see Electronic Supplementary Material Table S1: Fig. S1). They were selected to encompass a range of expected sources (e.g., tyre and non-tyre) and chemical properties which influence their fate in the environment (e.g., octanol-water partition coefficient—log *K*_*OW*_) (see Electronic Supplementary Material Table S2). Ultrapure water was 18.2 MΩ cm^−1^ quality. Water for method development and validation was collected from a series of four retention ponds that serve part of a 22-mile stretch of the trunk road network in Aberdeen and Aberdeenshire, North-East Scotland. The sampling location captures approximately 5 miles of dual carriageway and 2 miles of slip roads. Grab samples (500 mL) were collected from the inlet pipe of pond one (e.g., road runoff) and outlet pipe of the fourth pond for method development and validation.

### Sample preparation and UHPLC-MS/MS analysis

All water samples were prepared in triplicate and spiked with BPA-*d*_*8*_, 5-MTBR-*d*_*6*_, and 6PPD-quinone-*d*_*5*_ at a concentration of 1 µg/L. The mixture (1 mL) was vortexed before being filtered (13 mm, 0.45 µm, PTFE syringe filter) into a 1-mL glass vial ready for UHPLC-MS/MS analysis.

UHPLC-MS/MS analysis was carried out using an ACQUITY UPLC system (Waters Corporation, Manchester) coupled to a Xevo TQ-XS triple quadrupole mass spectrometer. Electrospray ionisation was performed in both positive (ESI+) and negative (ESI−) modes. The capillary voltage was 2.6 kV, and the low-mass resolution and high-mass resolution were 3.0 and 15.0, respectively. Nitrogen was used as the nebulisation and desolvation gas with a nebulising pressure of 7.0 bar, desolvation gas flow of 550 L min^−1^, a cone gas flow of 150 L h^−1^, and a gas temperature of 400 °C. In both positive and negative ionisation modes, ion energy 1 = 1.0 V and ion energy 2 = 2.0 V.

Two chromatographic methods were utilised to facilitate analysis of chemicals that ionise preferentially in ESI+ or ESI− ionisation modes. Separation was performed using an ACQUITY UPLC®BEH C18 column (1.7 µm 2.1 × 100 mm) with an injection volume of 10 µL. In both methods, a gradient elution was performed where mobile phase A was ultrapure water and mobile phase B was methanol. For chemicals analysed in ESI+, 0.1% formic acid and 5 mM ammonium formate were added to both mobile phases. For ESI−, both mobile phases contained 0.5 mM ammonium fluoride. The gradient elution was 16 min and 11.5 min for the ESI+ and ESI− methods, respectively (see Electronic Supplementary Material Table S3). Two multiple reaction monitoring (MRM) transitions (see Electronic Supplementary Material Table S4) were selected for each analyte, except for BLE and 6PPD-quinone-*d*_*5*_, BPA-d_8_, and 5-MBTR-d_6_ where only one was used. Only one MRM transition was monitored for BLE due to the lack of alternative MRM transitions with adequate sensitivity. The optimum cone voltage and collision energy were determined for all standards and deuterated standards (see Electronic Supplementary Material Table S4).

### Method development and assessment

Linearity of the UHPLC-MS/MS method was established using a nine-point calibration prepared in ultrapure water, and inlet and outlet retention pond water. Limits of quantification (LOQs) were determined as the lowest concentration at which the signal to noise ratio (*S:N*) was ≥ 10 and limits of detection (LODs) were taken as one-third of the LOQ. For those chemicals present in the inlet and outlet water samples, the LOQ was estimated based on their determined concentration and corresponding *S:N*. The concentrations of any chemicals present in blanks (ultrapure water) were subtracted from all samples. For these chemicals, the LOQ was set as the blank concentration (if the *S:N* was ≥10). Therefore, the concentration in samples (prior to adjustment) was required to be twice the blank concentration to be quantifiable.

Signal suppression during ESI was evaluated (Eq. 1).

1 $$\text{Signal supression }\left({\%}\right)=100-(\frac{Slope\; A}{Slope\; B} \times 100)$$

where *Slope A* is the slope of the calibration prepared in matrix (inlet or outlet water) and *Slope B* is the slope of the calibration prepared in ultrapure water.

Instrumental intra- and interday precision and accuracy were determined by performing triplicate injections of three concentrations of standard solutions (low, mid, and high) prepared in matrix within the same day and over three different days, respectively. The concentrations were 0.1, 1, and 10 µg/L for most compounds with the exception of BPA, HBCD, C-DMU, MBT, DMP, DEP, and BBP (1, 10, and 100 µg/L); 2-MTBT (2.5, 25, and 250 µg/L); and BT and 2-OHBT (10, 100, and 1000 µg/L) due to their lower sensitivity of detection. Standard solutions used to assess intraday precision and accuracy were kept in the autosampler maintained at 4 °C between injections. Standards were freshly prepared on different days to assess interday precision and accuracy.

The suitability of PTFE and PVDF syringe filters was assessed for the removal of particulate matter without the loss of analytes or their introduction by contamination. This was assessed in ultrapure water at two pH values (2 and 7) spiked at 1 µg/L (10 µg/L for BPA, HBCD, C-DMU, MBT, DMP, DEP, and BBP; 25 µg/L for 2-MTBT; and 100 µg/L for BT and 2-OHBT). Analysis was carried out in triplicate and the recovery under each condition determined by comparison to the corresponding standard (Eq. 2).

2 $$\text{Recovery}\;{\%}= \frac{{Response}_{A}}{{Response}_{B}} \times 100$$

Here, *Response*_*A*_ is the instrument response (peak area) of the filtered sample and *Response*_*B*_ is the response of the corresponding standard.

The recovery and trueness of the entire method (sample filtration and UHPLC-MS/MS analysis) were assessed at the low, mid, and high concentration levels previously described in both inlet and outlet water from the retention ponds. Recovery and trueness were determined (Eqs. 3 and 4).

3 $$\text{Recovery }\left(\%\right)= \frac{{(PA}_{A}- {PA}_{B})}{{PA}_{c}} \times 100$$

4 $$\text{Trueness }\left({\%}\right)= \frac{{(Conc}_{A}- {Conc}_{B})}{{Conc}_{c}} \times 100$$

Here, *PA*_*A*_ is the peak area of the spiked sample, *PA*_*B*_ is the peak area of the unspiked sample, and *PA*_*C*_ is the peak area of the corresponding standard solution. *Conc*_*A*_ is the determined concentration of spiked sample, *Conc*_*B*_ is the determined concentration of the unspiked sample, and *Conc*_*C*_ is the theoretical concentration of the corresponding standard.

Method detection limits (MDLs) and MQLs were calculated (Eqs. 5 and 6).

5 $$\text{MDL}= \frac{(\text{LOD }\times 100)}{Rec}$$

6 $$\mathrm{MQL}\;=\;\frac{(\mathrm{LOQ}\times100)}{Rec}$$

Here *Rec* is the average method recovery (%) from the low, mid, and high spike levels determined using Eq. 3.

### Monitoring

To demonstrate the application of the method, water samples were collected from the series of four retention ponds on four different days. During each sampling event, grab samples were collected from the inlet pipe of all four ponds as well as the outlet pipe of the final pond (see Electronic Supplementary Material Fig. S2). Each pond has a single inlet pipe and a single outlet pipe. The outlet pipe of pond 1 serves at the inlet pipe of pond 2 and so on. The outlet pipe of pond 4 discharges into a nearby watercourse (a small stream). Samples were collected in 250-mL polypropylene bottles. Rainfall data was taken from a nearby weather station approximately 6 miles from the sampling location (see Electronic Supplementary Material Fig. S3) [30].

### Quality assurance

Sample bottles were thoroughly rinsed with water and soaked overnight. They were then rinsed twice with tap water, twice with ultrapure water, and finally twice with LC-MS grade methanol. Field blanks of ultrapure water were used to determine any contamination from the sampling process. Procedural blanks were prepared for each batch of samples to quantify any contamination. Any contamination found in procedural blanks was subtracted from the sample results. All blanks and samples were prepared in triplicate.

## Results and discussion

### Method development

The protonated or deprotonated molecular ions of the plastic additive chemicals were monitored in ESI+ and ESI− mode, respectively. Most chemicals were ionised in ESI+ except for BPA, TBBPA, and HBCD which had greater response in ESI−. The two most sensitive MRM transitions were optimised and monitored for each analyte. For the deuterated surrogates, one MRM transition was optimised. Following optimisation of the MS/MS parameters for detection, a reversed phase chromatographic separation was developed using a water-methanol gradient and C18 BEH column. Two chromatography methods were utilised to maximise analyte sensitivity needed for direct injection analysis: one each for ESI− and ESI+. The mobile phase was based on similar analysis carried out by Rauert et al. [1]; however, the addition of ammonium buffers improved sensitivity and peak shape for several analytes. In particular, the addition of ammonium formate was necessary for the analysis of BT which suffered from low MS/MS response.

**Fig. 1:**
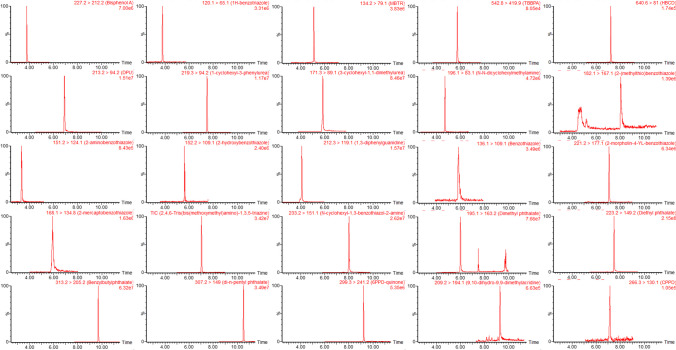
Example chromatograms for all 25 additives spiked in inlet retention pond water at 1 µg/L with the exceptions of BPA, HBCD, C-DMU, MBT, DMP, DEP, and BBP (10 µg/L); 2-MTBT (25 µg/L); and BT and 2-OHBT (100 µg/L)

The mobile phases used for analytes most sensitive in ESI+ contained 10 mM ammonium formate and 0.1% formic acid, whereas those most sensitive in ESI− had 0.5 mM ammonium fluoride added. The additives BPA, HBCD, C-DMU, 2-MTBT, 2-OHBT, BT, DMP, DEP, and BBP were included in the method at a higher concentration than the other analytes due to lower sensitivity. Analytes such as BPA have also been found to have low sensitivity in other LC-MS/MS methods, which is attributed to poor ionisation and fragmentation characteristics [13]. The studied additives have a broad range of chemicals properties (e.g., log *K*_*OW*_ range from 1.30 to 7.19; see Electronic Supplementary Material Table S2). This is reflected in their retention times ranging from 3.2 to 10.5 min in the ESI+ method and 3.8 to 7.2 min in the ESI− method (Fig. 1; see Electronic Supplementary Material Table S4).

**Fig. 2:**
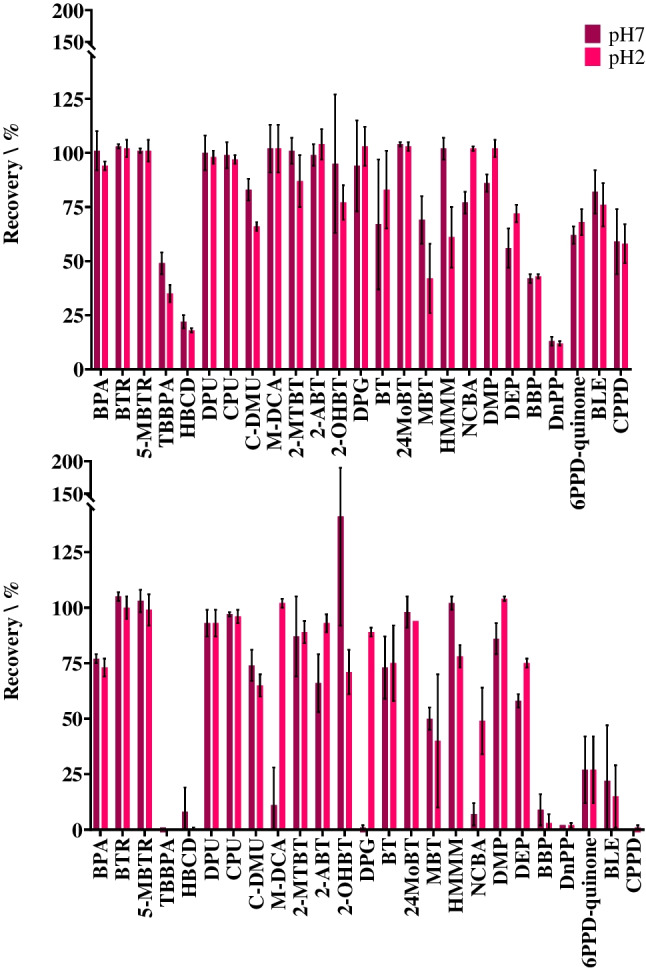
Recovery (%) of all 25 chemical additives from ultrapure water at pH 2 and pH 7 using two different types of syringe filters: PTFE (top) and PVDF (bottom)

Sensitivity of the instrument enabled sample analysis by direct injection following sample filtration to remove particulate matter. Selecting an appropriate filter membrane material is essential to minimise loss of target analytes and to ensure no unwanted contamination occurs. PTFE and PVDF were selected for investigation due to their suitability for analytes with a broad range of chemical properties [24]. Analyte recovery was assessed at pH 7 and pH 2. Overall, PTFE had higher average recoveries at both pH values, averaging 79% and 77% at pH 7 and pH 2, respectively, compared to 56% and 63% observed with the PVDF filter (Fig. 2). At pH 7, PTFE provided higher recoveries for the vulcanisation additives 2-ABT, MBT, DPG, and NCBA. Recoveries were 99%, 69%, 94%, and 77% using the PTFE filter, compared to the PVDF filter which gave recoveries of 66%, 50%, 1%, and 7%, respectively. Similarly, the two protective additives, BLE and CPPD, both showed higher recoveries for PTFE (82% and 59%) compared to PVDF (23% and 0%). 6PPD-quinone, a chemical of particular interest due to its reported toxicity in the environment, had recoveries of 62% and 27% for the PTFE and PVDF filters, respectively. Due to the ionisable nature of some of the analytes, recovery was also determined at pH 2 (see Electronic Supplementary Material Table S2). Overall, similarity in analyte recovery was observed in water at pH 2 and pH 7. Therefore, the sample preparation selected was no pH adjustment of the water and filtration using PTFE filter membranes. Low recovery (i.e., ≤50%) was still reported for TBBPA, HBCD, BBP, and DnPP due to their comparatively greater hydrophobicity and adsorption to the filter material. Several previous studies have used PTFE as a prefilter material prior to sample analysis for plastic additive chemicals by LC-MS/MS [12, 29]. In total, 10 chemicals were determined at least once in blank samples (instrumental, procedural, or field blanks) (Table 1). BPA and the phthalates were ubiquitous across all blanks. No notable differences in concentrations were found for instrumental, procedural, and field blanks demonstrating contamination was from the instrumental method over sample handling and processing. The maximum blank concentration was 9.4 µg/L for DEP (Table 1).

**Table 1:** Limits of detection (LODs) and limits of quantification (LOQs), linear range, and coefficient of determination (*r*^2^) for all additives in both inlet and outlet retention pond water samples

Use	Additive	Abbreviation	Inlet				Outlet				Max. blank conc. (µg/L)
			LOD (µg/L)	LOQ (µg/L)	Linear range (µg/L)	*r*^2^	LOD (µg/L)	LOQ (µg/L)	Linear range (µg/L)	*r*^2^	
Stabiliser	Bisphenol-A	BPA	0.069	0.23	0.23–100	0.9998	0.069	0.23	0.23–100	0.9994	0.23
	1H-Benzotriazole	BTR	0.0015	0.0050	0.0050–10	0.9985	0.0016	0.0052	0.0052–10	0.9997	-
	5-Methylbenzotriazole	5-MBTR	0.0015	0.0050	0.0050–10	0.9960	0.0014	0.0048	0.0048–10	0.9995	-
Flame retardant	Tetrabromobisphenol-A	TBBPA	0.0041	0.014	0.014–10	0.9996	0.0041	0.014	0.014–10	0.9999	0.014
	1,2,5,6,9,10-Hexabromocyclododecane	HBCD	0.015	0.050	0.050–100	0.9988	0.014	0.045	0.045–100	0.9986	-
Vulcanisation transformation product	*N,N*-Diphenylurea	DPU	0.0015	0.0050	0.0050–10	0.9932	0.0015	0.0049	0.0049–10	0.9992	-
Thermal degradation product	1-Cyclohexyl-3-phenylurea	CPU	0.0015	0.0050	0.0050–10	0.9991	0.0015	0.0048	0.0048–10	0.9978	-
	3-Cyclohexyl-1,1-dimethylurea	C-DMU	0.275	0.92	0.92–100	0.9974	0.28	0.92	0.92–100	0.9984	0.92
	*N,N*-Dicyclohexylmethylamine	M-DCA	0.0015	0.0050	0.0050–10	0.9986	0.0019	0.0065	0.0065–10	0.9998	-
Vulcanisation additive	2-(Methylthio)benzothiazole	2-MTBT	0.88	2.9	2.9–250	0.9997	0.73	2.4	2.4–250	0.9999	-
	2-Aminobenzothiazole	2-ABT	0.0032	0.011	0.011–10	0.9991	0.0039	0.013	0.013–10	>0.9999	-
	2-Hydroxybenzothiazole	2-OHBT	0.94	3.1	3.1–1000	0.9873	0.75	2.5	2.5–1000	0.9869	-
Vulcanisation accelerator	1,3-Diphenylguanidine	DPG	0.0015	0.0050	0.0050–10	0.9995	0.0014	0.0048	0.0048–10	0.9997	-
	Benzothiazole	BT	3.8	13	13–1000	0.9896	3.9	13	13–1000	0.9980	-
	2-(4-Morpholinyl)benzothiazole	24MoBT	0.013	0.044	0.044–10	0.9995	0.013	0.044	0.044–10	>0.9999	0.044
	2-Mercaptobenzothiazole	MBT	0.19	0.63	0.63–100	0.9902	0.23	0.75	0.75–100	0.9821	-
	2,4,6-Tris(bis(methoxymethyl)amino-1,3,5-triazine	HMMM	0.0015	0.0050	0.0050–10	0.9990	0.0015	0.0051	0.0051–10	>0.9999	-
	N-Cyclohexyl-1,3-benzothiazol-2-amine	NCBA	0.093	0.31	0.31–10	0.9980	0.093	0.31	0.31–10	0.9999	-
Plasticiser	Dimethylphthalate	DMP	0.22	0.74	0.74–100	0.9998	0.22	0.74	0.74–100	0.9999	0.74
	Diethylphthalate	DEP	2.8	9.4	9.4–100	0.9942	2.8	9.4	9.4–100	0.9962	9.4
	Benzylbutylphthalate	BBP	0.015	0.050	0.050–100	0.9993	0.015	0.051	0.051–100	0.9990	-
	Di-*n*-pentylphthalate	DnPP	0.034	0.11	0.11–10	0.9943	0.034	0.11	0.11–10	0.9999	0.11
Antioxidant transformation product	*N*-(1,3-Dimethylbutyl)-*N′*-phenyl-p-phenylenediamine quinone	6PPD-quinone	0.014	0.047	0.047–10	0.9987	0.014	0.047	0.047–10	0.9999	0.047
Protective additive	9,10-Dihydro-9,9-dimethylacridine	BLE	0.21	0.71	0.71–10	0.9979	0.21	0.71	0.71–10	0.9993	0.71
	*N*-Cyclohexyl-*N′*-phenyl-*p*-phenylenediamine	CPPD	0.017	0.058	0.058–10	0.9993	0.019	0.064	0.064–10	0.9936	0.31

### Instrument performance

The analytes determined in ESI− (BPA, TBBPA, and HBCD) were quantified against BPA-*d*_*8*_ (see Electronic Supplementary Material Table S4). Assignment of deuterated surrogate standards (5-MBTR-*d*_*6*_ or 6PPD-quinone-*d*_*5*_) for quantitation of analytes analysed in ESI+ mode was based on their similarity in recovery from environmental waters filtered through the PTFE filters. The performance of the UHPLC-MS/MS method was assessed for linearity and sensitivity for each analyte. Linearity was investigated for all 25 analytes over a nine-point concentration range (Table 1). All analytes had coefficients of determination (*r*^2^) values >0.993 except for 2-OHBT, BT, and MBT which exhibited *r*^2^ values <0.99. This is due to the absence of a corresponding deuterated surrogate that could adequately account for changes in response and their analysis was performed on a semi-quantitative basis. The LODs were in the range 1.5 × 10^−3^–3.8 µg/L and 1.4 × 10^−3^–3.9 µg/L in inlet and outlet water, respectively. LOQs for both inlet and outlet water were within the range 5.0 × 10^−3^–13 µg/L (Table 1).

The intraday and interday accuracy and precision was established by triplicate injection at low, mid, and high concentration levels during the same day, and across three different days, respectively. The intraday accuracy in inlet water samples averaged 100%, 107%, and 103% across all analytes for the low, mid, and high concentrations (range 64–144%). Similarly, the average intraday accuracy values in outlet water samples were 103%, 104%, and 95% (range 61–143%). Little difference was observed in comparison to the interday accuracy assessments for inlet or outlet water samples which were in the ranges 73–136% and 61–148%, respectively (Table 2). Similarity was also observed in precision determined on the same day for inlet (1–40%) and outlet water samples (1–43%) as well as between days (1–42% and 1–36% for inlet and outlet waters). An ideal range for accuracy is 90–110% with precision values <10%. Although the results of many analytes fell within these ranges, several did not. This is most problematic for those analytes which showed poor precision. In particular 2-OHBT, BLE, and CPPD had several precision results ≥30% (Table 2).

**Table 2:** Intra- and interday precision (%) and accuracy (%) and signal suppression (%) for all additives in inlet and outlet retention pond water. The low, mid, and high concentrations were 0.1, 1, and 10 µg/L respectively with the exceptions of BPA, HBCD, C-DMU, MBT, DMP, DEP, and BBP (1, 10, and 100 µg/L); 2-MTBT (2.5, 25, and 250 µg/L); and BT and 2-OHBT (10, 100, and 1000 µg/L)

Additive	Inlet													Outlet												
	Intraday accuracy (%)			Intraday precision (%)			Interday accuracy (%)			Interday precision (%)			% Signal suppression	Intraday accuracy (%)			Intraday precision (%)			Interday accuracy (%)			Interday precision (%)			% Signal suppression
	Low	Mid	High	Low	Mid	High	Low	Mid	High	Low	Mid	High		Low	Mid	High	Low	Mid	High	Low	Mid	High	Low	Mid	High	
BPA	93	100	99	12	6	5	80	99	99	6	5	3	−10	89	111	110	12	4	3	80	106	104	3	8	8	−7
BTR	119	101	104	3	6	7	114	114	103	14	7	2	2	112	102	90	5	2	3	104	106	100	16	5	8	5
5-MBTR	129	107	106	11	5	8	125	117	108	15	5	5	−3	132	102	87	6	5	2	134	98	94	8	2	7	−8
TBBPA	127	79	82	11	6	12	112	91	90	4	11	15	−34	113	93	94	11	10	7	89	91	91	12	9	9	−19
HBCD	80	71	64	14	10	14	82	89	81	17	21	27	32	61	87	83	32	14	10	54	94	87	22	16	12	22
DPU	96	121	109	16	11	4	73	106	107	31	6	8	−9	80	121	96	25	3	6	75	115	99	11	14	1	−10
CPU	111	133	125	3	3	5	92	128	111	22	6	14	2	109	126	105	6	3	3	83	112	98	25	11	2	−1
C-DMU	80	110	100	11	4	4	79	115	102	25	8	3	1	100	110	96	5	7	6	71	115	101	28	8	2	6
M-DCA	82	81	88	20	8	11	91	90	95	13	4	8	−24	113	98	101	19	14	21	124	107	108	9	7	11	5
2-MTBT	-	107	107	-	16	3	-	102	108	-	11	5	3	-	84	83	-	8	4	-	87	91	-	8	11	−14
2-ABT	119	91	85	6	8	5	130	104	94	5	10	4	−17	92	99	103	8	12	6	95	108	107	15	6	8	5
2-OHBT	68	119	117	40	31	17	94	134	107	30	20	8	13	141	119	90	12	30	12	148	121	102	32	19	2	−7
DPG	73	102	108	3	2	5	75	130	116	6	11	20	1	93	100	103	11	6	5	101	117	98	12	22	5	−3
BT	-	144	138	-	5	5	-	124	113	-	36	31	−18	-	143	119	-	12	4	-	141	100	-	14	12	−15
24MoBT	102	110	116	15	6	8	103	114	107	12	2	9	0	108	112	98	6	2	3	105	104	100	4	5	2	−1
MBT	-	78	96	-	13	7	-	126	83	-	12	7	−18	-	80	99	-	16	14	-	120	87	-	16	23	3
HMMM	112	132	125	8	2	6	99	125	111	20	8	14	2	132	125	115	16	8	6	118	115	106	24	18	10	3
NCBA	-	127	125	-	4	9	-	121	108	-	9	12	1	-	118	107	-	4	1	-	102	101	-	11	5	−2
DMP	97	101	87	15	7	1	83	107	97	18	10	10	−7	86	87	80	4	5	3	92	103	92	4	10	9	−6
DEP	-	131	108	-	6	6	-	127	104	-	11	8	1	-	102	91	-	8	4	-	112	92	-	2	5	−10
BBP	105	114	91	2	7	1	99	112	88	3	1	1	−6	91	103	85	3	1	1	87	110	89	6	6	3	−4
DnPP	-	108	102	-	4	5	-	114	102	-	6	5	2	-	100	94	-	7	5	-	110	101	-	3	4	−1
6PPD-quinone	94	114	110	6	7	2	81	108	102	5	2	6	2	114	103	102	7	1	3	90	104	101	18	4	3	2
BLE	-	126	122	-	31	14	-	136	113	-	42	13	4	-	100	81	-	13	6	-	86	88	-	12	13	−28
CPPD	109	66	73	39	21	26	110	85	82	28	41	33	41	94	81	73	12	43	26	105	61	89	26	36	36	51

‘-’ indicates no value available as the concentration was below the MQL

Hou et al. [13] reported comparatively better intraday and interday precisions at their low concentration level (which is 10 times higher than this study) for the majority of the same analytes studied with values of 1–18% and 1–27% for BPA, BTR, 5MBTR, 2-ABT, DPG, 24MoBT, HMMM, NCBA and 2-OHBT prepared in solvent (methanol) over matrix. The low concentration for BPA, one of the less sensitive analytes, was 10 µg/L in both studies. Hou et al. presented intraday and interday precision values of 18% and 10%, respectively, for BPA [13]. This study presented slightly lower values, with intraday precision of 12% for both inlet and outlet, respectively, and interday precision of 6% in the inlet and 3% in the outlet. However, it should be noted that the method described by Hou et al. [13] had a range of other analytes including pesticides, pharmaceuticals, and personal care products which can result in trade-offs in method performance to achieve satisfactory performance for a broad range of chemicals. To minimise variability in the instrument response for analytes such as 2-OHBT, BLE, and CPPD in environmental samples a calibration was performed for every 15 samples analysed.

Signal suppression (and enhancement) during ESI caused by the sample matrix was measured. The analyte with the most enhanced signal was TBBPA in inlet water (−34% suppression) and the most suppressed was CPPD in outlet water (51% suppression) (Table 2). This broad range of suppression is not uncommon for the environmental analysis of trace organic chemicals by LC-MS/MS [23, 31]. Notable differences were also observed in the suppression of the same analyte between water samples. For example, M-DCA had suppression of −24% and 5% in inlet water and outlet water, respectively. This is likely due to difference in the dissolved organic composition of inlet and outlet water samples. Those analytes that have corresponding deuterated surrogates available (BPA, 5-MBTR, and 6PPD-quinone) had low signal suppressions in the range from −10 to 2% in both water samples compared to several analytes such as TBBPA, HBCD, M-DCA, BLE, and CPPD (Table 2). Therefore, signal suppression cannot be accurately accounted for by the available deuterated surrogates in the method. This demonstrates the need to prepare calibrations within matrix where only a few deuterated surrogates are available for multi-residue analysis.

### Method performance

**Fig. 3:**
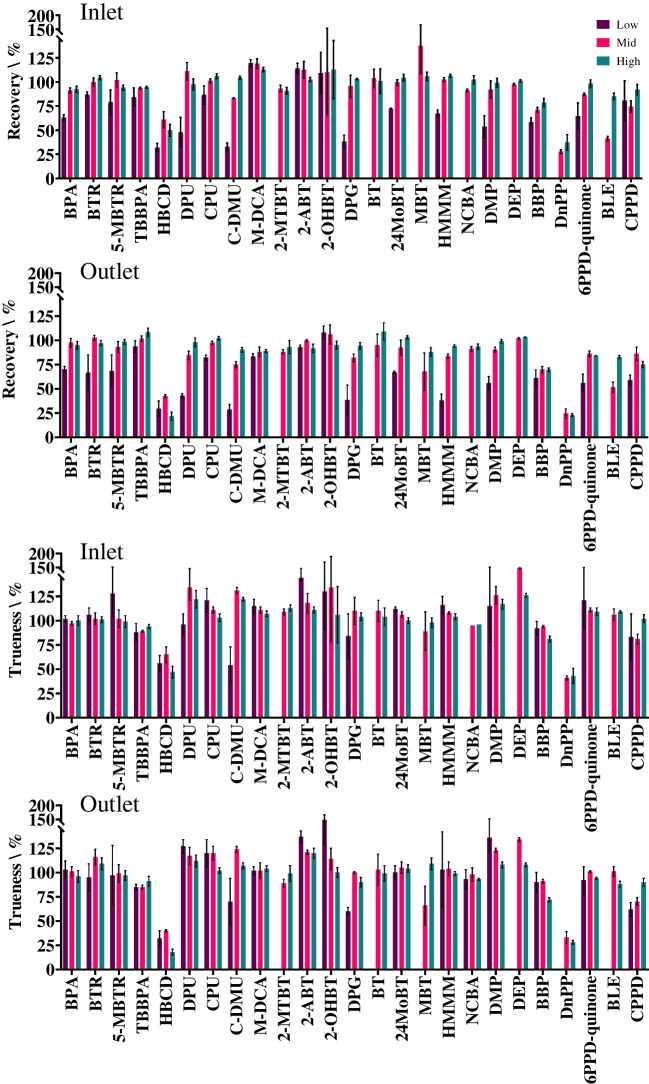
Recovery (%) and trueness (%) for all 25 chemical additives spiked at three different concentrations. The low, mid, and high concentrations were 0.1, 1, and 10 µg/L respectively with the exceptions of BPA, HBCD, C-DMU, MBT, DMP, DEP, and BBP (1, 10, and 100 µg/L); 2-MTBT (2.5, 25, and 250 µg/L); and BT and 2-OHBT (10, 100, and 1000 µg/L). From top to bottom, the graphs show recovery from inlet retention pond water, recovery from outlet retention pond water, trueness of inlet retention pond water determinations, and trueness of outlet retention pond water determinations

Analyte recovery of the entire analytical method (sample filtration and UHPLC-MS/MS analysis) was assessed at three concentration levels in inlet and outlet retention pond water. Due to the variety of chemicals included in the method, it is unsurprising that a degree of variation was observed (Fig. 3). Recovery from inlet water ranged from 28 ± 2.1% for DnPP to 138 ± 29% for MBT (both at the mid spike concentration). Other than the loss of hydrophobic analytes to the PFTE filters previously described, their adsorption to particulates within the water samples prior to filtration will result in losses.

The cumulative average recovery for the 25 analytes was 72%, 92%, and 95% for the low, mid, and high concentrations, respectively. In the outlet water samples, the recoveries were in the range of 22 ± 4.2% for HBCD to 109 ± 8.7% for BT (both at the high spike level) with averages for all analytes being 63%, 84%, and 88% over the three concentration ranges. The recoveries >100% is attributed to signal enhancement during ESI (Table 2). Recoveries and variation in recoveries were comparable to previous direct injection LC-MS/MS methods for analysis of environmental waters, albeit for different analytes and matrices [32, 33]. Trueness of the method was also established in inlet and outlet pond water at the three different concentration levels. Overall, values ranged from 18 ± 3.5% to 148 ± 19%. This variation is attributed to the lack of deuterated surrogates available for the analytes included in the method. Nevertheless, most chemicals fell within the 80–120% range with relative standard deviations ≤20% (Fig. 3).

The inlet and outlet MDLs ranged from 1.3 × 10^−3^ µg/L for M-DCA to 3.7 µg/L for BT (Table 3). The corresponding MQLs were in the range 4.3 × 10^−3^–13 µg/L. Most analytes were quantifiable at concentrations <1 µg/L, with some exceptions being C-DMU, 2-MTBT, 2-OHBT, BT, DEP, and BLE. The reported MQLs are adequate to monitor the majority of the additive chemicals at or below their current freshwater PNEC values contained within the NORMAN database (Table 3) [15]. Although 6PPD-quinone does not have an established PNEC, the MQLs of 0.056 µg/L and 0.062 µg/L for inlet and outlet water were below its lowest acute toxicity threshold reported in the literature (0.095 µg/L) [16]. However, it is important to consider that the future establishment of a PNEC for 6PPD-quinone, once adequate toxicity data is available, will likely be below the reported MQL of the method. Nevertheless, the MQLs here are sufficiently sensitive to monitor additive chemicals at concentrations previously reported in the aquatic environment [1, 12, 18, 19, 34]. Lower MDLs and MQLs are reported in the literature [1, 12, 13, 34]; however, they utilise SPE as a sample preconcentration step. This newly developed method has the advantages of fast and lower cost sample preparation whilst still being able to determine environmentally relevant concentrations of plastic additive chemicals.

**Table 3:** Method detection limits (MDLs) and method quantification limits (MQLs) and freshwater PNEC values (when given) for all 25 additives in inlet and outlet retention pond water. PNEC values were taken from the NORMAN database [15]

Additive	Inlet		Outlet		Lowest PNEC freshwater (µg/L)
	MDL (µg/L)	MQL (µg/L)	MDL (µg/L)	MQL (µg/L)	
BPA	0.084	0.28	0.079	0.26	0.24
BTR	0.0015	0.0052	0.0018	0.0058	19
5-MBTR	0.0016	0.0055	0.0017	0.0055	150
TBBPA	0.0046	0.015	0.0041	0.014	0.064
HBCD	0.032	0.11	0.043	0.14	0.0016
DPU	0.0018	0.0059	0.0020	0.0066	0.7
CPU	0.0015	0.0051	0.0015	0.0052	3.2
C-DMU	0.37	1.3	0.43	1.4	-
M-DCA	0.0013	0.0043	0.0022	0.0074	1.52
2-MTBT	0.96	3.2	0.81	2.7	0.69
2-ABT	0.0029	0.010	0.0041	0.014	1
2-OHBT	0.85	2.8	0.73	2.4	14
DPG	0.0019	0.0063	0.0020	0.0067	1.05
BT	3.7	12	3.8	13	240
24MoBT	0.014	0.048	0.015	0.050	-
MBT	0.15	0.51	0.29	0.97	0.76
HMMM	0.0016	0.0054	0.0021	0.0070	-
NCBA	0.096	0.32	0.10	0.34	0.093
DMP	0.27	0.91	0.27	0.91	192
DEP	2.9	9.5	2.8	9.2	73
BBP	0.022	0.072	0.023	0.076	5.2
DnPP	0.11	0.35	0.14	0.48	0.088
6PPD-quinone	0.017	0.056	0.019	0.062	-
BLE	0.34	1.1	0.32	1.1	0.17
CPPD	0.021	0.071	0.026	0.087	-

### Application to retention pond samples

**Fig. 4:**
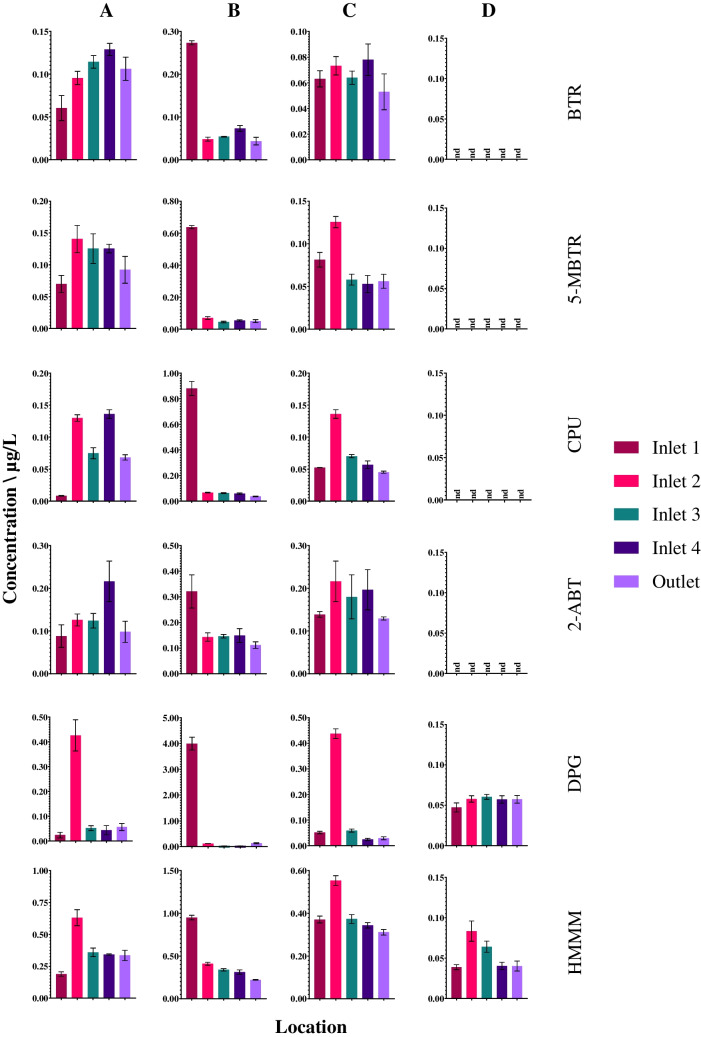
The concentrations of the seven chemical additives which were detected with a frequency ≥75% (*n* = 16) during four different sampling events (**A**, **B**, **C**, and **D**) of four roadside retention ponds in series located in North-East Scotland. The scale on the *y*-axis varies between sampling events and chemicals due to differences in the concentrations observed. nd, not detected

The developed method was applied to samples collected from a series of four retention ponds that serve a 22-mile stretch of the trunk road network in North-East Scotland during four different sampling events of differing rainfall conditions (A, B, C, and D). Sampling event A was conducted on a dry day, following a period of low rainfall (≤4.4 mm/day in the preceding 12 days) and sampling event B was conducted during heavy rainfall, but after a period of low rainfall (≤4.4 mm/day in at least the preceding 21 days). Sampling was conducted approximately 1 hour after the start of the storm. Sampling C was conducted the day after heavy rainfall (13.8 mm after an 11-day period of no rain) and sampling D was conducted on a dry day with low rainfall the preceding week (6.2 mm), but high levels of consistent rain during the month prior (up to 31.6 mm/day) (see Electronic Supplementary Material Fig. S2). Grab samples were collected from the inlet of each pond (ponds 1, 2, 3, and 4) and from the outlet of the final pond (pond 4) which then discharges into a small stream. In total, 9 of the 25 studied additive chemicals were detected at least once in the water samples (Fig. 4). They are all tyre-related additives and have log *K*_*OW*_ values ≤3.8 (see Electronic Supplementary Material Table S2). More hydrophobic chemicals may be present and associated with particles within the water. Both DPG and HMMM were detected with 100% frequency (*n* = 20). BTR, 5-MBTR, CPU, and 2-ABT were detected in 75% of samples (*n* = 16). The remaining chemicals found in samples (6PPD-quinone, M-DCA, and 2-OHBT) were detected once. The detected chemicals are in agreement with research by Peter et al. [34].

It should be noted that the sampling approach does not account for the water residence time within the retention ponds and limits the conclusions that can be drawn regarding the removal of the chemicals by the ponds. However, relatively stable flow through the ponds in the 10 days prior to sampling event A (rainfall ranged from 0 to 4.4 mm/day) enables some understanding of additive chemical behaviour within the ponds. BTR, 5-MBTR, and 2-ABT showed similar concentrations at each of the sampling locations. The range of concentrations observed were 0.060–0.13 µg/L, 0.070–0.14 µg/L, and 0.088–0.13 µg/L, respectively (Fig. 4), suggesting little or no removal of these chemicals by the ponds. Interestingly, CPU, DPG, and HMMM showed an increase in concentration between the inlets of pond 1 and pond 2. Most notably, DPG increased from 0.024 to 0.43 µg/L during sampling event A. It is postulated that this is caused by its leaching from TRWPs in pond 1. Previous research has found TRWPs are retained in ponds by settling into sediment with greatest concentrations found near the inlet compared to the pond outlet [35, 36]. Laboratory-based investigations have also found tyre additive chemicals including DPG and HMMM readily leach from tyre particles in water [37].

Sampling event B was conducted on a day during heavy rainfall (13.8 mm) following 11 days without any rainfall. Despite high flow at the inlet pond 1, the greatest concentrations of all chemicals were observed here. For example, DPG was present at 4.0 ± 0.25 µg/L which is ~10 times higher than any other sample. Notably, 6PPD-quinone was also present at 0.13 ± 0.006 µg/L. Both M-DCA and 2-OHBT were also detected in this sample at 0.063 ± 0.002 µg/L and 4.3 ± 0.9 µg/L, respectively. Previous studies have found higher concentrations of both TRWPs and additive chemicals during storm events [18]. At the time of sampling, the increased flow had not reached the other ponds, and the concentrations observed were typical of ‘dry weather’ observations (Fig. 4). Sampling event C occurred the day after sampling event B and there was no noticeable impact on water quality through the retention ponds (i.e., no increased tyre chemical concentration) following the rain event. The concentrations observed were similar to sampling event A. This demonstrates the buffering effect of the retention ponds during rainfall on additive chemical concentrations before the water is discharged.

Sampling event D was undertaken on a dry day following a period of heavy rainfall. A total of 43 and 147 mm of rain was recorded in the previous 10 and 21 days prior to sampling taking place (see Electronic Supplementary Material Fig. S2). Here, only HMMM and DPG were present at 0.047–0.060 µg/L and 0.038–0.083 µg/L, respectively. It is hypothesised that the heavy rainfall caused TRWPs present in the ‘upstream’ drainage system (e.g., roadside filter drains) to be washed into and through the retention ponds which would normally leach additive chemicals.

It is important to consider the possible risk of plastic additives within the ponds themselves and the significance of concentrations in the outlet which discharges to the wider aquatic environment. Commonly, the PNEC which indicates the concentration below which no adverse effects are expected is used to assess the environmental risk. Of those chemicals which have an available PNEC (Table 3), only DPG (*n* = 1) exceeded this concentration. The current PNEC of 1.05 µg/L was exceeded in inlet 1 during sampling event B (4.0 ± 0.25 µg/L). Most significantly, in the sample where 6PPD-quinone was detected (0.13 ± 0.006 µg/L in inlet 1 of sampling event B), it exceeded its lowest reported acute toxicity threshold of 0.095 µg/L [16]. Although this *LC*_*50*_ value was determined for juvenile Pacific salmon and not directly relevant to the retention ponds in question, the findings categorise 6PPD-quinone as ‘very highly toxic’ to aquatic organisms [38]. This demonstrates that further studies on plastic additive chemicals in retention ponds are needed. Such studies can be supported with this newly developed analytical method. Improved understanding of the presence and fate of these chemicals in retention ponds will facilitate the optimisation of pond design and operation for their removal.

## Conclusions

A direct injection UHPLC-MS/MS method was successfully developed and validated for the detection and quantification of plastic additives in road runoff and retention ponds. Direct injection reduced the sample preparation requirements, lowering the likelihood of loss or contamination, as well as decreasing the sample preparation time. The elimination of a pre-concentration step has not been detrimental to the sensitivity with MQLs in the low µg/L range and below current freshwater PNECs of most chemicals. MQLs ranged from 4.3 × 10^−3^ to 13 µg/L. The lack of deuterated surrogates available for the studied analytes required matrix matched calibrations to reduce the influence of signal suppression caused by other substances present within the samples. The recovery and trueness of the method were assessed and the majority of analytes falling within the range of 80–120%. Application of the method to samples collected from retention ponds during four sampling events showed the presence of eight chemicals at quantifiable levels, including 6PPD-quinone. DPG was detected in one sample at a concentration 4.0 ± 0.25 µg/L, higher than its current PNEC value. This demonstrated the suitability of the method for its intended purpose and supports the need for further work to monitor these potentially toxic chemicals in drainage systems.

## Supplementary Information

Below is the link to the electronic supplementary material.

Supplementary file1 (DOCX 663 KB)
