# Supplementary material for: Development of a straightforward direct injection UHPLC-MS/MS method for quantification of plastic additive chemicals in roadside retention ponds
Source: Anal Bioanal Chem. 2024 Nov 25;417(2):389–403. doi: 10.1007/s00216-024-05657-3 (PMC11698802; doi:10.1007/s00216-024-05657-3)
Supplement: Supplementary file 1 — Supplementary file1 (DOCX 663 KB) [file 216_2024_5657_MOESM1_ESM.docx]

# Supplementary Material

**Development of a straightforward direct injection UHPLC-MS/MS method for quantification of plastic additive chemicals in roadside retention ponds**

Katie McKenzie^a^, Angela Pllu^b^, Iain Campbell^b^, Linda A. Lawton^a^, Bruce Petrie^a^

^a^School of Pharmacy, Applied Sciences and Public Health, Robert Gordon University, Aberdeen, AB10 7GJ

^b^Balfour Beatty plc, UK Construction Services – Motherwell, Scotland, ML1 4WQ

This supplementary material contains three figures and four tables outlining the chemical structures and properties of the studied plastic additives, retention pond layout, rainfall data and the UHPLC gradient programme and MS/MS parameters of the developed method.

***Fig. S1*** Chemical structure for all plastic additives included in the study. Structures were produced using ChemDraw software.


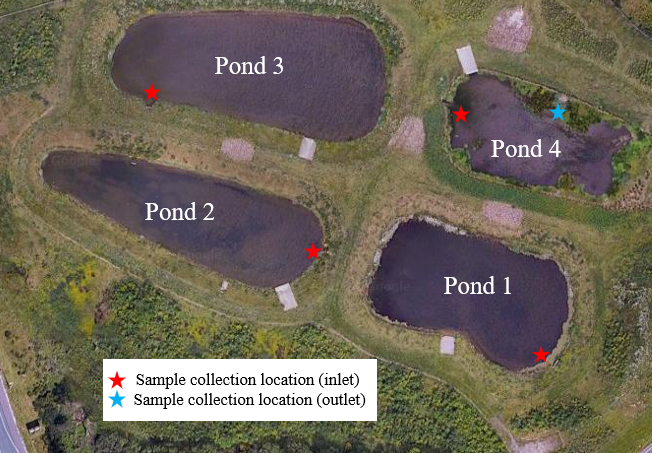


**Fig. S2** Layout of the studied retention ponds and the five sample collection points from inlet/outlet pipes. The road is approximately 50 m from the ponds at its closest point. Image adapted from google maps [1]

**Fig. S3** Rainfall levels in the 21 days prior to each of the four sampling events. Adapted from data provided by the Scottish Environmental Protection Agency from their weather station located approximately six miles from sampling location [2].

**Table S1** The properties of the 25 plastic additives and three deuterated surrogates included in the analysis.

| **Use** | **Additive** | **Abbreviation** | **Molecular Weight \ g/mol** | **Formula** | **CAS** | **Supplier** |
| --- | --- | --- | --- | --- | --- | --- |
| Stabiliser | Bisphenol-A | BPA | 228.29 | C_15_H_16_O_2_ | 80-05-7 | Sigma |
|  | 1H-benzotriazole | BTR | 119.12 | C_6_H_5_N_3_ | 95-14-7 | Fisher |
|  | 5-methylbenzotriazole | 5-MBTR | 133.15 | C_7_H_7_N_3_ | 136-85-6 | Sigma |
| Flame retardant | Tetrabromobisphenol-A | TBBPA | 543.9 | C_15_H_12_Br_4_O_2_ | 79-94-7 | TCI |
|  | 1,2,5,6,9,10-hexabromocyclododecane | HBCD | 641.7 | C_12_H_18_Br_6_ | 3194-55-6 | Sigma |
| Transformation product | N,N-diphenylurea | DPU | 212.25 | C_13_H_12_N_2_O | 102-07-8 | TCI |
| Thermal degradation product | 1-cyclohexyl-3-phenylurea | CPU | 218.29 | C_13_H_18_N_2_O | 886-59-9 | Sigma |
|  | 3-cyclohexyl-1,1-dimethylurea | C-DMU | 170.25 | C_9_H_18_N_2_O | 31468-12-9 | Sigma |
|  | N,N-dicyclohexylmethylamine | M-DCA | 195.34 | C_13_H_25_N | 7560-83-0 | Fisher |
| Vulcanisation additive | 2-(methylthio)benzothiazole | 2-MTBT | 181.3 | C_8_H_7_NS_2_ | 615-22-5 | TCI |
|  | 2-aminobenzothiazole | 2-ABT | 150.2 | C_7_H_6_N_2_S | 136-95-8 | Fisher |
|  | 2-hydroxybenzothiazole | 2-OHBT | 151.9 | C_7_H_5_NOS | 934-34-9 | Fisher |
| Vulcanisation accelerator | 1,3-diphenylguanidine | DPG | 211.26 | C_13_H_13_N_3_ | 102-06-7 | Fisher |
|  | Benzothiazole | BT | 135.19 | C_7_H_5_NS | 95-16-9 | Sigma |
|  | 2-(4-Morpholinyl)benzothiazole | 24MoBT | 220.29 | C_11_H_12_N_2_OS | 4225-26-7 | Sigma |
|  | 2-mercaptobenzothiazole | MBT | 167.3 | C_7_H_5_NS_2_ | 149-30-4 | Sigma |
|  | 2,4,6-tris(bis(methoxymethyl)amino-1,3,5-triazine | HMMM | 390.44 | C_15_H_30_N_6_O_6_ | 3089-11-0 | TRC |
|  | N-cyclohexyl-1,3-benzothiazol-2-amine | NCBA | 232.35 | C_13_H_16_N_2_S | 28291-75-0 | Sigma |
| Plasticizer | Dimethylphthalate^*^ | DMP | 194.18 | C_10_H_10_O_4_ | 131-11-3 | Sigma |
|  | Diethylphthalate^*^ | DEP | 222.24 | C_12_H_14_O­ | 84-66-2 | Sigma |
|  | Benzylbutylphthalate^*^ | BBP | 312.4 | C_19_H_20_O_4_ | 85-68-7 | Sigma |
|  | di-n-pentylphthalate^*^ | DnPP | 306.4 | C_18_H_26_O_4_ | 131-18-0 | Sigma |
| Antioxidant | N-(1,3-dimethylbutyl)-N′-phenyl-p-phenylenediamine quinone | 6PPD-quinone | 298.4 | C_18_H_22_N_2_O_2_ | 2754428-18-5 | LGC |
| Protective additive | 9,10-dihydro-9,9-dimethylacridine | BLE | 209.29 | C_15_H_15_N | 6267-02-3 | TCI |
|  | N-cyclohexyl-N-phenylenediamine | CPPD | 266.4 | C_18_H_22_N_2_ | 101-87-1 | LGC |
| Deuterated Surrogates | Bisphenol A–d8 | BPA–d8 | 236.34 | C_15_H_16_O_2_ | 92739-58-7 | TRC |
|  | N-(1,3-dimethylbutyl)-N′-phenyl-p-phenylenediamine quinone-d5 | 6PPD-quinone-d5 | 303.42 | C_18_H_17_D_5_N_2_O_2_ | - | LGC |
|  | 5-methylbenzotriazole-d6 | 5-MBTR-d6 | 143.22 | C_7_H_11_N_3_ | 1246820-65-4 | TRC |

**Table S2** Physicochemical properties of the 25 plastic additives included in the method.

| **Additive** | **Predicted Log *K_OW_*** | **Predicted *pKa*** | **Inlet (pH 8.08)** | | | **Outlet (pH 9.30)** | | |
| --- | --- | --- | --- | --- | --- | --- | --- | --- |
|  |  |  | **Log *D_OW_*** | **Ionisation \ %** | **Dominant speciation** | **Log *D_OW_*** | **Ionisation \ %** | **Dominant speciation** |
| BPA | 4.04 | 9.78 and 10.39 | 4.04 | 1.97 | Neutral | 3.91 | 26.4 | Neutral |
| BTR | 1.30 | 8.63 | 1.22 | 22.13 | Neutral | 0.83 | 82.51 | -1 |
| 5-MBTR | 1.81 | 8.85 | 1.74 | 14.55 | Neutral | 1.39 | 73.87 | -1 |
| TBBPA | 7.12 | 6.57 and 7.18 | 4.76 | 99.65 | -2 | 3.23 | 100.00 | -2 |
| HBCD | 7.19 | NA | 7.19 | NA | NA | 7.19 | NA | NA |
| DPU | 2.40 | NA | 2.40 | NA | NA | 2.40 | NA | NA |
| CPU | 2.90 | 13.57 | 2.90 | 0.00 | Neutral | 2.90 | 0.01 | Neutral |
| C-DMU | 1.11 | NA | 1.11 | NA | NA | 1.11 | 1.11 | NA |
| M-DCA | 3.79 | 11.82 | 0.48 | 99.98 | +1 | 1.31 | 99.70 | +1 |
| 2-MTBT | 3.43 | 1.13 | 3.43 | 0.00 | Neutral | 3.43 | 3.43 | Neutral |
| 2-ABT | 1.97 | 3.58 | 1.97 | 0.00 | Neutral | 1.97 | 1.97 | Neutral |
| 2-OHBT | 2.49 | 10.27 | 1.99 | 0.64 | Neutral | 1.95 | 1.95 | Neutral |
| DPG | 3.13 | 9.38 | 1.37 | 95.28 | +1 | 2.06 | 54.87 | +1 |
| BT | 2.11 | 2.28 | 2.11 | 0.00 | Neutral | 2.11 | 0.00 | Neutral |
| 24MoBT | 2.69 | 5.12 | 2.98 | 6.97 | Neutral | 2.67 | 55.44 | Neutral |
| MBT | 2.89 | 3.63 | 1.58 | 100.0 | -1 | 1.58 | 100.00 | -1 |
| HMMM | 2.59 | 1.13 | 2.59 | 0.00 | Neutral | 2.59 | 0.00 | Neutral |
| NCBA | 4.07 | 3.34 | 4.07 | 0.00 | Neutral | 4.07 | 0.00 | Neutral |
| DMP | 1.98 | NA | 1.98 | NA | NA | 1.98 | NA | NA |
| DEP | 2.69 | NA | 2.69 | NA | NA | 2.69 | NA | NA |
| BBP | 5.03 | NA | 5.01 | NA | NA | 5.03 | NA | NA |
| DnPP | 5.52 | NA | 5.52 | NA | NA | 5.52 | NA | NA |
| 6PPD-quinone | 3.24 | 11.77 | 3.24 | 0.02 | Neutral | 3.24 | 0.34 | Neutral |
| BLE | 4.12 | 1.28 | 4.12 | 0.00 | Neutral | 4.12 | 0.00 | Neutral |
| CPPD | 4.69 | 7.07 and 0.58 | 4.65 | 8.83 | Neutral | 4.68 | 0.58 | Neutral |

NA = Not available and represents chemicals in which no ionisable species are present.

**Table S3** Gradient programme for the UHPLC-MS/MS methods

| **Ionisation** | **Time \ min** | **Flow Rate \ mL/min** | **%A** | **%B** | **Curve** |
| --- | --- | --- | --- | --- | --- |
| ESI+ | 0.0 | 0.35 | 95 | 5 | Initial |
|  | 0.5 |  | 95 | 5 | 0.6 |
|  | 9.0 |  | 20 | 80 | 0.6 |
|  | 11.0 |  | 5 | 95 | 0.6 |
|  | 12.0 |  | 5 | 95 | 0.6 |
|  | 12.1 |  | 95 | 5 | 0.6 |
|  | 16.0 |  | 95 | 5 | 0.6 |
| ESI- | 0.0 | 0.35 | 95 | 5 | Initial |
|  | 0.5 |  | 95 | 5 | 0.6 |
|  | 2.5 |  | 40 | 60 | 0.6 |
|  | 7.0 |  | 5 | 95 | 0.6 |
|  | 8.0 |  | 5 | 95 | 0.6 |
|  | 8.1 |  | 95 | 5 | 0.6 |
|  | 11.5 |  | 95 | 5 | 0.6 |

**Table S4** UHPLC-MS/MS parameters for all compounds and internal standards, including the MRM transitions, retention time, cone voltage, collision energy, and the assigned internal standard.

| **Additive** | **Retention time \ min** | **ESI** | **Precursor ion \ m/z** | **Product ion \ m/z** | **Cone voltage \ V** | **Collison energy \ eV** | **Deuterated surrogate** |
| --- | --- | --- | --- | --- | --- | --- | --- |
| BPA | 3.8 | - | 227.2 | 133.1 | 34 | 28 | BPA-*d_8_* |
|  |  |  |  | 212.2 | 34 | 18 |  |
| BTR | 3.8 | + | 120.1 | 65.1 | 20 | 19 | 5-MBTR-*d_6_* |
|  |  |  |  | 92.1 | 20 | 13 |  |
| 5-MBTR | 5.1 | + | 134.2 | 106.1 | 20 | 15 | 5-MBTR-*d_6_* |
|  |  |  |  | 79.1 | 20 | 16 |  |
| TBBPA | 5.7 | - | 542.8 | 419.9 | 38 | 40 | BPA-*d_8_* |
|  |  |  |  | 445.9 | 40 | 33 |  |
| HBCD | 7.2 | - | 640.6 | 79.0 | 18 | 16 | BPA-*d_8_* |
|  |  |  |  | 81.0 | 18 | 16 |  |
| DPU | 6.9 | + | 213.2 | 94.2 | 16 | 18 | 6PPD quinone-*d_5_* |
|  |  |  |  | 77.1 | 16 | 26 |  |
| CPU | 7.5 | + | 219.3 | 94.2 | 20 | 14 | 5-MBTR-*d_6_* |
|  |  |  |  | 77.1 | 20 | 20 |  |
| C-DMU | 5.8 | + | 171.3 | 89.1 | 20 | 13 | 6PPD quinone-*d_5_* |
|  |  |  |  | 72.1 | 20 | 15 |  |
| M-DCA | 4.7 | + | 196.1 | 55.1 | 20 | 21 | 6PPD quinone-*d_5_* |
|  |  |  |  | 83.1 | 20 | 22 |  |
| 2-MTBT | 8.1 | + | 182.1 | 167.1 | 20 | 18 | 6PPD quinone-*d_5_* |
|  |  |  |  | 123.1 | 20 | 19 |  |
| 2-ABT | 3.2 | + | 151.2 | 109.1 | 20 | 19 | 6PPD quinone-*d_5_* |
|  |  |  |  | 124.1 | 20 | 26 |  |
| 2-OHBT | 5.6 | + | 152.2 | 109.1 | 20 | 14 | 5-MBTR-*d_6_* |
|  |  |  |  | 119.1 | 20 | 13 |  |
| DPG | 4.1 | + | 212.3 | 119.1 | 20 | 25 | 5-MBTR-*d_6_* |
|  |  |  |  | 94.1 | 20 | 26 |  |
| BT | 5.8 | + | 136.1 | 109.1 | 20 | 20 | 5-MBTR-*d_6_* |
|  |  |  |  | 77.1 | 20 | 22 |  |
| 24MoBT | 7.0 | + | 221.2 | 177.1 | 20 | 21 | 5-MBTR-*d_6_* |
|  |  |  |  | 150.1 | 20 | 26 |  |
| MBT | 5.9 | + | 168.1 | 134.8 | 20 | 24 | 6PPD quinone-*d_5_* |
|  |  |  |  | 123.9 | 20 | 20 |  |
| HMMM | 7.0 | + | 391.3 | 359.3 | 20 | 7 | 5-MBTR-*d_6_* |
|  |  |  |  | 177.2 | 20 | 22 |  |
| NCBA | 7.8 | + | 233.2 | 151.1 | 20 | 17 | 5-MBTR-*d_6_* |
|  |  |  |  | 55.2 | 20 | 21 |  |
| DMP | 6.0 | + | 195.1 | 133.1 | 18 | 22 | 6PPD quinone-*d_5_* |
|  |  |  |  | 163.2 | 18 | 8 |  |
| DEP | 7.5 | + | 223.2 | 149.2 | 18 | 18 | 6PPD quinone-*d_5_* |
|  |  |  |  | 177.1 | 18 | 8 |  |
| BBP | 9.7 | + | 313.2 | 205.2 | 22 | 7 | 6PPD quinone-*d_5_* |
|  |  |  |  | 91.1 | 22 | 12 |  |
| DnPP | 10.5 | + | 307.2 | 149.0 | 20 | 13 | 6PPD quinone-*d_5_* |
|  |  |  |  | 219.2 | 20 | 9 |  |
| 6PPD-quinone | 9.2 | + | 299.2 | 241.2 | 10 | 27 | 6PPD quinone-*d_5_* |
|  |  |  |  | 215.2 | 10 | 18 |  |
| BLE | 9.3 | + | 209.2 | 194.1 | 27 | 16 | 5-MBTR-*d_6_* |
| CPPD | 7.1 | + | 266.3 | 130.1 | 18 | 30 | 6PPD quinone-*d_5_* |
|  |  |  |  | 223.2 | 18 | 22 |  |
| BPA-*d_8_* | 3.8 | - | 235.2 | 220.2 | 34 | 21 | - |
| 5-MBTR-*d_6_* | 5.1 | + | 140.2 | 85.1 | 20 | 16 | - |
| 6PPD quinone-*d_5_* | 9.2 | + | 304.3 | 220.2 | 10 | 18 | - |

**References**

1. Google Maps. (2024) In: Google Maps. https://www.google.co.uk/maps/place/ Accessed 27 Jun 2024

2. SEPA Scottish Environmental Protection Agency: Rainfall data for Scotland. https://www2.sepa.org.uk/rainfall. Accessed 21 Jun 2024
